# 异基因造血干细胞移植后Wernicke脑病1例

**DOI:** 10.3760/cma.j.issn.0253-2727.2022.01.019

**Published:** 2022-01

**Authors:** 勇 张, 晓牧 谈, 合冰 周

**Affiliations:** 1 首都医科大学附属北京潞河医院血液科，北京 101149 Department of Hematology, Beijing Luhe hospital, Capital Medical University, Beijing 101149, China; 2 首都医科大学附属北京潞河医院神经科，北京 101149 Department of Neurology, Beijing Luhe Hospital, Capital Medical University, Beijing 101149, China

患者，女，40岁，2020年1月诊断为“骨髓增生异常综合征EB-2（WPSS评分3分）”，分别于2020年1月、2020年3月和2020年5月应用地西他滨去甲基化治疗。移植前骨髓原始粒细胞占0.020，微小残留病（MRD）0.77％，WT1基因定量18.658％，NPM1-A基因突变定量1.244％。预处理方案：阿糖胞苷4 g·m^−2^·d^−1^，移植前10 d（−10 d）、−9 d，地西他滨20 mg·m^−2^·d^−1^，−10 d～−6 d，白消安3.2 mg·kg^−1^·d^−1^，−8 d～−6 d，环磷酰胺1.8 g·m^−2^·d^−1^，−5 d、−4 d，兔抗人胸腺细胞球蛋白2.5 mg·kg^−1^·d^−1^，−5 d～−2 d，司莫司汀250 mg/m^2^，−3 d，移植物抗宿主病（GVHD）预防方案：西罗莫司、霉酚酸酯联合短程甲氨蝶呤。患者于2020年8月18日行单倍型造血干细胞移植（女供母，HLA 6/12，AB+供A+），回输供者外周血干细胞单个核细胞（MNC）9.63×10^8^/kg，CD34^+^细胞3.84×10^6^/kg。移植后11 d（+11 d）粒细胞及血小板植活。+3 d患者发生呕血，给予禁食水、抑酸治疗。患者高热，血培养、病毒、细菌、真菌筛查均为阴性，给予广谱抗生素抗感染治疗后体温未降。+8 d，患者出现皮疹。+9 d，患者发生上腹部烧灼感伴腹泻，考虑急性GVHD，加用甲泼尼龙2 mg·kg^−1^·d^−1^，体温降至正常。+13 d，患者血脂明显升高（甘油三酯38.18 mmol/L，总胆固醇8.65 mmol/L）。患者间断恶心、呕吐伴上腹不适，+14 d，患者出现乳糜血（甘油三酯41.70 mmol/L，总胆固醇7.49 mmol/L），停西罗莫司（此时西罗莫司浓度17.7 µg/L），改为环孢素A治疗GVHD，甘油三酯降至3.80 mmol/L，总胆固醇降至6.15 mmol/L。+25 d，患者出现嗜睡，恶心、呕吐好转，血巨细胞病毒、EB病毒DNA阴性，环孢素A浓度165.5 µg/L。+26 d，患者昏睡，定向力、计算力障碍、四肢肌力下降，腰穿脑脊液压力100 mmH_2_O，氯114.3 mmol/L，葡萄糖8.30 mmol/L，蛋白0.55 g/L。脑脊液细菌培养、真菌培养及抗酸杆菌均阴性，脑脊液神经免疫病检测：血清和脑脊液中AQP4、MOG、MBG抗体均为阴性。脑脊液和外周血病毒PCR筛查均阴性，脑脊液病原体二代测序（NGS）阴性。TMA筛查阴性。头颅平扫+增强磁共振成像（MRI）：双侧颞叶深部（海马区）及室管膜下区、左侧颞叶皮层多发异常信号并下丘对侧斑片明显强化灶。右侧小囊幕增厚并明显强化。结合患者近1个月进食差，伴有恶心、呕吐、腹泻，考虑不除外Wernicke脑病，给予维生素B_1_ 200 mg每8 h 1次治疗，+27 d患者神志转清，肢体肌力明显好转。患者维生素B_1_<1.0 µg/L，明确诊断为Wernicke脑病，继续补充维生素B_1_治疗。复查头颅MRI：双侧颞叶（及海马区）及室管膜下区多发异常信号并双侧下丘、右侧小脑幕明显强化灶，较前部分病变信号明显减低，范围减小。+30 d，复查骨髓完全缓解（CR），MRD阴性，NPM1基因突变阴性，短串联重复序列-聚合酶链反应（STR-PCR）完全供者型。+36 d，间断谵妄，给予奥氮平对症治疗后好转，+50 d，患者谵妄好转，停用奥氮平。随访至移植后8个月，患者无明显神经、精神症状及体征，环孢素A血浓度50 µg/L，原发病处于完全缓解状态，MRD阴性，STR-PCR完全供者型。头颅MRI：双侧颞叶（及海马区）及室管膜下区多发异常信号并双侧下丘脑、右侧小脑幕病变强化灶明显减低，范围明显缩小。

讨论：Wernicke脑病是一种急性神经系统综合征，由维生素B_1_缺乏引起，通常表现以眼球运动障碍，步态障碍或共济失调和精神状态改变为特征。MRI是诊断Wernicke脑病强有力的工具，典型表现为双侧对称的T2加权像、弥散加全权像的异常高信号影，受累部位常见于丘脑、第三脑室、第四脑室、乳头体、中脑导水管周围、桥脑和延髓。移植后患者出现长时间纳差或禁食、频繁呕吐及腹泻等维生素B_1_缺乏风险时，应给予维生素B_1_预防Wernicke脑病。

